# Deformation-based morphometry: a sensitive imaging approach to detect radiation-induced brain injury?

**DOI:** 10.1186/s40644-024-00736-1

**Published:** 2024-07-18

**Authors:** Carole Brunaud, Samuel Valable, Gwenn Ropars, Fatima-Azzahra Dwiri, Mikaël Naveau, Jérôme Toutain, Myriam Bernaudin, Thomas Freret, Marianne Léger, Omar Touzani, Elodie A. Pérès

**Affiliations:** 1 Université de Caen Normandie, CNRS, Normandie Université, ISTCT UMR6030, GIP Cyceron, Caen, F-14000 France; 2 Université de Caen Normandie, CNRS, INSERM, Normandie Université, UAR 3408/US50, GIP Cyceron, Caen, F-14000 France; 3 Université de Caen Normandie, INSERM, Normandie Université, COMETE UMR-S 1075, GIP Cyceron, Caen, F-14000 France

**Keywords:** Radiotherapy, Brain, Magnetic resonance imaging, Deformation-based morphometry, Macrostructural changes

## Abstract

**Background:**

Radiotherapy is a major therapeutic approach in patients with brain tumors. However, it leads to cognitive impairments. To improve the management of radiation-induced brain sequalae, deformation-based morphometry (DBM) could be relevant. Here, we analyzed the significance of DBM using Jacobian determinants (JD) obtained by non-linear registration of MRI images to detect local vulnerability of healthy cerebral tissue in an animal model of brain irradiation.

**Methods:**

Rats were exposed to fractionated whole-brain irradiation (WBI, 30 Gy). A multiparametric MRI (anatomical, diffusion and vascular) study was conducted longitudinally from 1 month up to 6 months after WBI. From the registration of MRI images, macroscopic changes were analyzed by DBM and microscopic changes at the cellular and vascular levels were evaluated by quantification of cerebral blood volume (CBV) and diffusion metrics including mean diffusivity (MD). Voxel-wise comparisons were performed on the entire brain and in specific brain areas identified by DBM. Immunohistology analyses were undertaken to visualize the vessels and astrocytes.

**Results:**

DBM analysis evidenced time-course of local macrostructural changes; some of which were transient and some were long lasting after WBI. DBM revealed two vulnerable brain areas, namely the corpus callosum and the cortex. DBM changes were spatially associated to microstructural alterations as revealed by both diffusion metrics and CBV changes, and confirmed by immunohistology analyses. Finally, matrix correlations demonstrated correlations between JD/MD in the early phase after WBI and JD/CBV in the late phase both in the corpus callosum and the cortex.

**Conclusions:**

Brain irradiation induces local macrostructural changes detected by DBM which could be relevant to identify brain structures prone to radiation-induced tissue changes. The translation of these data in patients could represent an added value in imaging studies on brain radiotoxicity.

**Supplementary Information:**

The online version contains supplementary material available at 10.1186/s40644-024-00736-1.

## Background

Radiotherapy (RT) is performed annually on several hundreds of thousands of patients with brain tumors. Although it undeniably improves patient survival, cranial RT inevitably leads to adverse effects due to irradiation of the healthy brain tissue. Radiation toxicities appear at different times following RT: acute effects from days to weeks with fatigue and variable psycho-cognitive changes, then early effects from 1 to 6 months after RT, especially manifest and progressive cognitive deficits and finally late effects can appear several months or years after RT corresponding to permanent cognitive disabilities [1]. Cranial RT induces cognitive decline associated with a reduction in the quality of life of long-surviving patients [2, 3]. Several preclinical studies evidenced that brain irradiation causes vascular lesions, white matter necrosis, demyelination, inflammation, neurogenesis alterations and neurodegeneration [4, 5].

To improve the diagnosis and the management of RT-induced brain sequalae, sensitive tools are needed. Magnetic resonance imaging (MRI), by its non-invasive and multiparametric nature, represents the technique of choice to monitor and predict RT-induced cerebral alterations [6].

Diffusion MRI by probing the movement of water molecules assesses the tissue microstructure, and seems promising because changes in diffusion metrics in gray or white matter are associated to tissue loss and demyelination [7]. Vascular MRI is also relevant to study vascularization changes (blood volume, blood flow) and blood brain barrier permeability that are known to be involved in the pathophysiology of radiation-induced delayed effects [8]. Although there is an association between cognitive and radiation-induced brain injury, their causative relationship remains unclear [9].

Even if advanced MRI seems very interesting for detection of brain radiotoxicity, it is not easy to use and interpret in clinical routine. It is therefore necessary to offer analyses based on routinely used MRI sequences. The macrostructural changes can be evaluated by anatomical MRI based on T1- or T2-weighted imaging to perform volumetry or morphometry investigations. Two computational approaches are mainly used to identify progressive volume loss and local vulnerability of brain areas: voxel-based morphometry (VBM) and deformation-based morphometry (DBM).

The VBM requires to segment the brain into gray matter (GM), white matter (WM) and cerebrospinal fluid (CSF), warping with a rigid transformation the segmented images to a template space and smoothing, and then, the volume of GM, WM, CSF can be statistically analyzed between groups [10].

The DBM is based on deformation fields obtained by non-linear registration of brain images. The DBM identifies structural differences throughout the whole brain from the gradients of non-linear deformation fields required to registration of the brain subject on a reference template which is generated from the study’s pool of brains, a brain atlas or a derived template generator. In DBM, the statistical analyses are not performed on the registered voxels but on the deformation fields used to register them [11]. These deformation fields enable to identify differences in the relative positions of cerebral structures and determine the local morphological changes. Generally, they are associated with Jacobian determinants (JD), which characterize the change in volume at each voxel between the template and the sample brain [10]. Contrary to VBM, DBM can be performed on T1- or T2-weighted images from different research laboratories or medical centers, an issue that is very convenient for multicentric projects [12].

DBM is increasingly used in several brain diseases in both rodents [13–15] and human situations [12, 16, 17]. But so far, only fragmentary studies using DBM approach are reported on radiation-induced brain injury [18, 19].

From a cerebral radiotoxicity model based on the whole-brain irradiation in the rat, in which we previously showed a late radiation-induced brain atrophy [20], we aimed to 1/ assess the relevance of DBM approach to detect brain local macrostructural alterations induced by irradiation and 2/ investigate the potential relationships between macrostructural and microstructural changes in the most vulnerable cerebral structures identified by DBM.

## Methods

### Animals

All animal investigations were performed on 6-month-old male Wistar rats (Janvier Labs) under the current European directive (2010/63/EU). Ethical approval was obtained from the regional ethics committee (CENOMEXA) and the French Ministère de l’Enseignement Supérieur, de la Recherche et de l’Innovation (APAFIS #00919). This study, based on multiparametric MRI, was longitudinally performed to get closer to the temporality of appearance of the symptoms described by the patients: early (from 1 to 3 months) and late (around 6 months) effects. At each time point, extra animals were also included for immunohistochemical analyses.

### Brain irradiation

Before irradiation, rats were anesthetized with 5% isoflurane in 30% O_2_ / 70% N_2_O (3 min) and maintained during the brain irradiation procedure (10 min) with 2.5% isoflurane. Rats were exposed to fractionated whole-brain irradiation (WBI) distributed equally over 3 consecutive days (3 × 10 Gy). We retained this radiation dose to have translational relevance by ensuring that the pathophysiology induced after cerebral irradiation is relatively similar between rats and humans WBI as previously described [20]. WBI was performed using a small animal dedicated irradiator with the inboard scanner (X-RAD 225Cx, Precision X-ray). To ensure spatial accuracy of the deposited dose and homogenous irradiation in the brain, the WBI paradigm was defined from a treatment planning system (TPS) through the use of SmART-Plan software [21] (Additional Fig. 1). The details of WBI protocol were: voltage = 225 kV, intensity = 13 mA, energy = 80 keV, dose rate = 3.3 Gy/min, copper filtration (thickness = 1 mm), diameter circular collimator = 25 mm. The animals were then monitored daily until the end of the study (up to 6 months post-WBI).

**Fig. 1:**
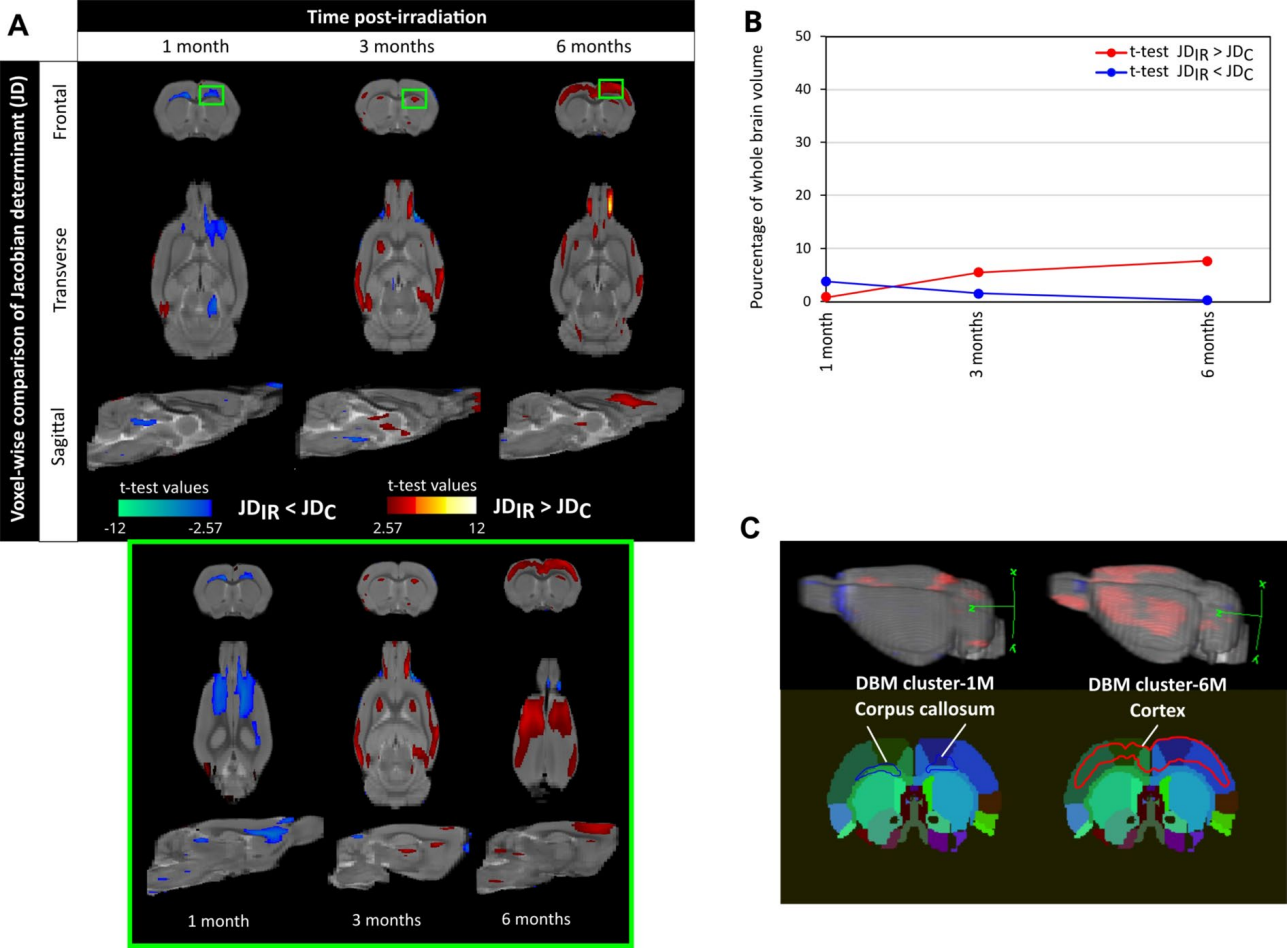
Deformation-based morphometry reveals local macrostructural changes over time induced by brain irradiation in cerebral structures. (**A**) Voxel-wise comparison of Jacobian determinant (JD) between control and irradiated groups (noted C and IR respectively) at 1, 3 and 6 months after irradiation (Student’s t-test with significant threshold set at *p* < 0.01). The JD was quantified from T2w images that were registered on a rat brain template performed on control rats at the specific post-irradiation time studied. The statistically different voxels are located on the rat brain template (T2w image). The blue clusters correspond to significant t-test when JD values of irradiated group are lower than those of the control group. The red clusters correspond to significant t-test when JD values of irradiated group are higher than those of the control group. The green box depicts a zoom of the clusters at 1, 3 and 6 months after irradiation. (**B**) Longitudinal quantification of proportion of significant voxels relative to the whole brain volume. Blue line represents the blue clusters (significant t-test for JD _irradiated_ < JD _control_) and red line corresponds to red clusters (significant t-test for JD _irradiated_ > JD _control_). (**C**) From deformation-based morphometry (DBM), the significant large clusters identified at 1 month (DBM-1 M in blue) and 6 months (DBM-6 M in red) after irradiation were registered on a rat brain atlas to identify the most vulnerable brain areas. The cluster DBM-1 M is located in the corpus callosum and the cluster DBM-6 M is located in the cortex. The number of animals for the control group is *N* = 6–9 rats and for the irradiated group is *N* = 6–7 rats

### MRI acquisitions

MRI was performed on a 7 Tesla magnet (Pharmascan^®^, Bruker). A cross coil configuration was used (volume/surface coil, Bruker). For all MRI experiments, rats under volatile anesthesia (2.5% isoflurane in 30% O_2_ / 70% N_2_O) were placed in the prone position, their heads secured with ear and tooth bars. The respiration (60 cycles/min) and body temperature (37 °C) were maintained throughout the duration of MRI examinations. After a localizer sequence, a multiparametric MRI examination was performed for each animal (from 1 up to 6 months after WBI). The total duration of an examination was 45 min per animal. Sequential MRI exams were acquired in the following order: (1) anatomic MRI, (2) diffusion MRI and (3) vascular MRI. The main parameter of MRI sequences as described below.

#### Anatomic MRI

T2-weighted imaging was based on T2w-RARE sequence: acceleration factor of 4; repetition time (TR)/echo time (TE_eff_) = 5000/30 msec; average = 1; repetitions = 4; 64 contiguous slices; field of view (FOV) = 32 × 32 mm; spatial resolution = 0.125 × 0.125 × 0.5 mm^3^; acquisition time = 16 min.

#### Diffusion MRI

Diffusion Tensor Imaging (DTI) was acquired from Single Shot-Echo Planar Imaging (SE-EPI) sequence: TR/TE = 2200/37 msec; 30 directions and 2 b values = 0 and 1000 s/mm^2^; A0 images = 5; average = 1; repetitions = 3; 32 contiguous slices; FOV = 32 × 32 mm; spatial resolution = 0.167 × 0.167 mm x 1 mm^3^; acquisition time = 15 min.

#### Vascular MRI

T2* imaging using Gradient Echo-Echo Planar Imaging (GE-EPI) performed before and after intravenous injection of a contrast agent in caudal vein. T2* images were acquired prior and 4 min after intravenous injection of P904^®^ (200 µmol/kg, Chematech): TR/TE = 20 000/13 msec; average = 1; repetitions = 6; 32 contiguous slices; FOV = 32 × 32 mm; spatial resolution = 0.290 × 0.290 × 1 mm^3^; acquisition time = 2 min.

### MRI analyses

#### Deformation based morphology from T2w images (anatomic MRI)

DBM consists of two steps: a non-linear registration and computation of Jacobian determinant. In this study, Advanced Normalization Tools (ANTs) software (version 2.2.0) was used for the different registrations [22]. The step-by-step analysis is described in Additional Fig. 2A.

**Fig. 2:**
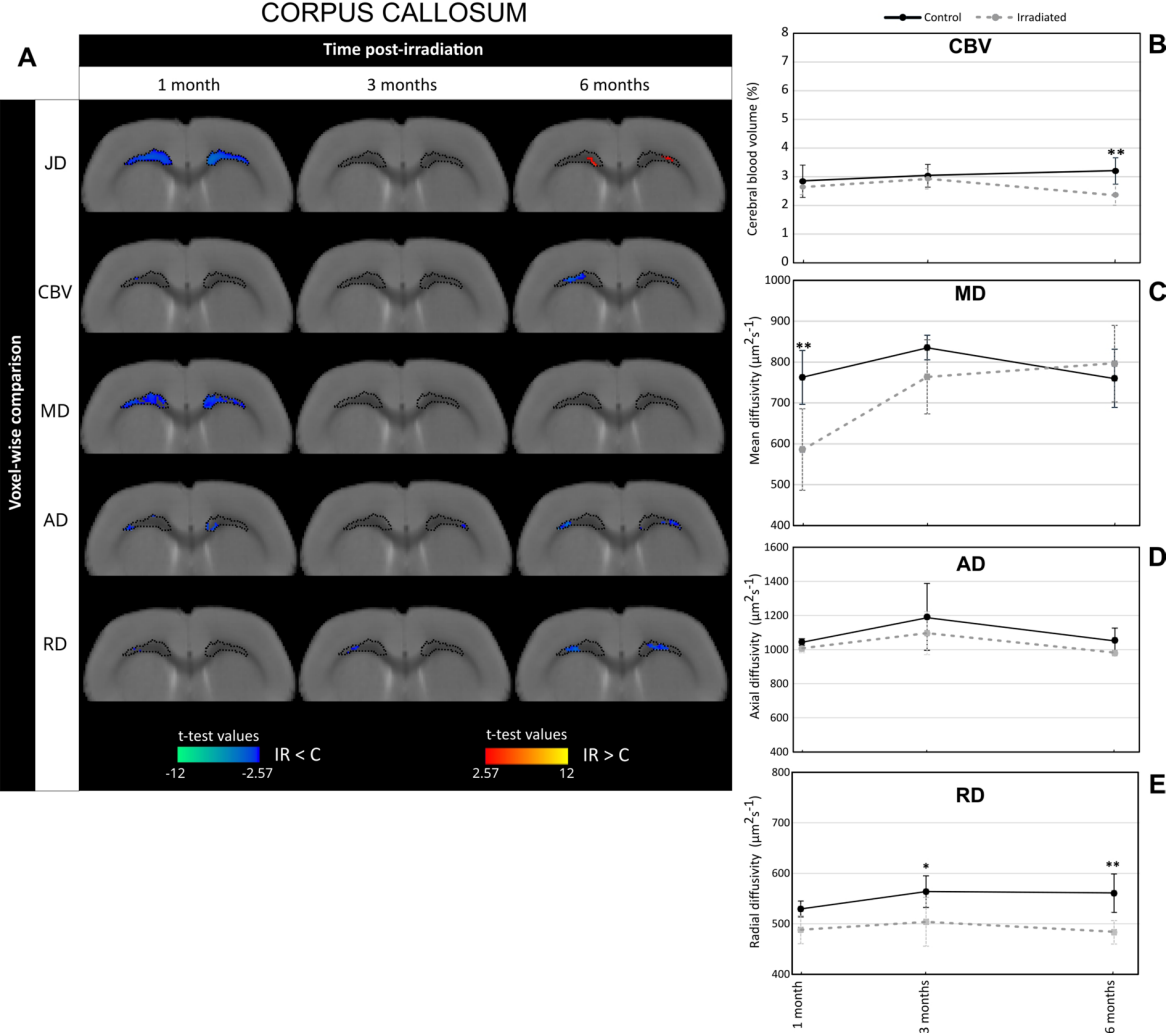
Irradiation induces changes in cerebral blood volume and MRI-derived diffusion parameters in the corpus callosum. (**A**) Voxel-wise comparison of Jacobian determinant (JD), cerebral blood volume (CBV), mean diffusivity (MD), axial diffusivity (AD) and radial diffusivity (RD) in the corpus callosum between control and irradiated groups (noted C and IR respectively) at different times after whole-brain irradiation (Student’s t-test with significant threshold set at *p* < 0.01). The macrostructural changes highlighted by DBM in the corpus callosum at 1 month post-irradiation are demarcated on the T2w images by dotted lines. The statistically different voxels are located on the rat brain template (T2w image). The blue clusters correspond to significant t-test when MRI parameter values of irradiated group are lower than those of the control group. The red clusters correspond to significant t-test when MRI parameter values of irradiated group are higher than those of the control group. (**B-E**) Quantification of CBV (**B**), MD (**C**), AD (**D**) and RD (**E**) in the corpus callosum at 1, 3 and 6 months after whole-brain irradiation. Black line corresponds to the control group and gray line to the irradiated group. The number of animals for the control group is *N* = 6 rats and for the irradiated group is *N* = 4–6 rats. Mean ± SD, two-way ANOVA (group and time effects) followed by Fisher’s LSD test: * *p* < 0.05, ** *p* < 0.01

##### Masking

Before registration, the T2w images of each animal were masked to remove non-brain tissue using BrainSuite17a software (version 2) [23]: the delineation was semi-automatic and contour errors were corrected manually on a slice-by-slice basis. From these brain masks, the whole-brain volume was measured using ImageJ software (version 1.52k) [24].

##### Registration

Firstly, the registration involved an intensity inhomogeneity correction with the “N4BiasFieldCorrection function” of ANTs on T2w images. Then, a non-linear registration was applied on each masked and corrected image on the reference template with the script “antsRegistration”. This function involved: a rigid transformation with a similarity metrics by mutual information, an affine transformation with a similarity metrics by mutual information and a SyN transformation with a similarity metrics by cross-correlation, and a linear interpolation when the warped images were saved.

The reference template (Mean-Control template) corresponded to average of the T2w images of all control animals at each time point to exclude the potential ageing effect.

##### Jacobian determinant (JD)

Lastly, JD maps were created on ANTs with the script “CreateJacobianDeterminantImage”. The JD maps represent tensor deformations between each rat brain MRI and the template for each voxel. The definition of the Jacobian (J), here we used log (Jacobian), is an unitless measure defined by the ratio of volumes, defined by:

$$\log J\left( x \right) = V\left( {\Phi \left( x \right)} \right)/V\left( x \right)$$

where V represents the volume operation and x corresponds to a small object. If $\varPhi$ causes expansion, then $J\left(x\right)>0$.

Thereby, JD quantifies the local expansion ($J\left(x\right)>0)$or contraction $(J\left(x\right)<0)$between the warped image and Mean-Control template.

##### Rat brain atlas

To identify the brain structures associated to major morphometry changes revealed by DBM analysis, a homemade MRI-derived neuroanatomical rat brain atlas was used. This is based on manually segmentation and annotation of cerebral areas from a stereotactic atlas of brain in Wistar rat: 238 structures have been identified and grouped on 29 areas.

Then, the rat brain atlas was registered to the homemade rat brain template with the PMOD software (version 3.0) (www.pmod.com), with a rigid registration.

#### Cerebral blood volume from T2* images (vascular MRI)

The step-by-step analysis of cerebral blood volume (CBV) maps is described in Additional Fig. 2B.

##### Pre*-*processing

The contrast agent P904^®^ which belongs to the USPIO family has been used to calculate cerebral blood volume at equilibrium. As the stationary state of P904^®^ in brain vessels being reached from 4 min after intravenous injection, we performed T2* imaging using Gradient Echo-Echo Planar Imaging (GE-EPI) before and 4 min after intravenous injection of P904^®^. The cerebral blood volume (CBV) maps were generated from T2* images acquired before and after contrast agent injection using a homemade macro from ImageJ software based on the Steady-State method (SS-ΔR2*) [25]. The stationary approach allows quantitative estimation of CBV maps with the following Eq. [26]:

$$CBV\left( \% \right)=\frac{3}{{4\pi }}.\left( {\frac{{\Delta R{2^*}}}{{\gamma .\Delta \chi .{\text{Bo}}}}} \right)$$

Where CBV reflecting the amount of blood in brain tissue is expressed in % (corresponding to ml blood/100 ml tissue); $\varDelta {R2}^{*}$is the signal variation on T2* images obtained before and after contrast agent injection; $\varDelta$ is the increase in the magnetic susceptibility difference between the extra- and intravascular compartments (nonrationalized units) induced by the presence of the contrast agent in the vasculature; $\text{B}\text{o}$ is the main magnetic field (T); and γ is the gyromagnetic ratio of protons.

##### Registration

Firstly, T2w images, acquired with the same resolution that T2* images, were semi-automatically masked using the BrainSuite software and they were registered on our homemade rat brain template with a non-linear registration using the ANTs software. The characterization of registration included a similarity metrics by mutual information and a linear interpolation when the warped image was saved. Then, the transformation matrix was applied on CBV maps with the script “antsApplyTransforms” on ANTs software to obtain warped CBV maps.

#### Diffusion metrics from DTI images (diffusion MRI)

The step-by-step analysis of diffusion maps is described in Additional Fig. 2C.

##### Pre-processing

DTI images were processed by the Paravision software (version 6.0, Bruker) to obtain the various resulting diffusion metrics maps: mean diffusivity (MD), axial diffusivity (AD), and radial diffusivity (RD) [27].

##### Registration

Before this registration, the non-diffusion-weighted T2 images (A0 images), were semi-automatically masked using the BrainSuite software and they were registered on our homemade rat brain template using ANTs software. The transformation matrix obtained was then applied to the different diffusion parameter maps.

#### Extraction of values of parametric maps

The mean values of different MRI parameters metrics (JD, CBV, MD, AD, RD) were extracted in significant clusters identified by DBM (see “Statistical analyses”) using ImageJ software.

### Immunohistological analyses

Post-mortem analyses were performed at 1, 3 and 6 months after WBI. Rats received a subcutaneous injection of 0.05 mg/Kg of buprenorphine (Buprécare^®^, Axience) 30 min before being deeply anesthetized with 5% isoflurane in 30% O_2_ and 70% N_2_O. The animals underwent a transcardiac perfusion with cold heparinized saline solution and the brains were removed and immediately frozen in isopentane and stored at -80 °C. Serial coronal brain sections of 20 μm thickness were obtained using a cryostat.

To depict brain vascularization, astrocytes and myelin on brain slices, primary antibodies used were anti-collagen-IV (1:100; 134,001; Southern Biotech), anti-glial fibrillary acidic protein (GFAP) (1:500; Z0334; Dako) and anti-myelin basic protein (MBP) (1:500 ab40370; Abcam), respectively. Before the staining, non-specific binding sites were blocked with 3% of bovine serum albumin (BSA) and 0.1% Tween/0.5% Triton in phosphate buffered saline (PBS) solution for 2 h at room temperature. The brain slices were then incubated with the primary antibodies in 1% BSA/0.1% Tween/0.5% Triton in PBS at 4 °C overnight. The staining was revealed by a fluorochrome-conjugated secondary antibodies Alexa Fluor 488 (1:500; Abcam) co-incubated with Hoechst 33,342 (1:1000; Sigma-Aldrich) for nuclei staining. Brain sections were examined under a Leica DMi8 fluorescence microscope (x10 magnification).

### Statistical analyses

All data are presented as mean ± standard deviation. The voxel-wise comparisons between experimental groups were performed with Student’s t-test using SPM12 on MATLAB (The MathWorks, Natick, MA) with p-value < 0.01 (corrected for multiple comparisons using family-wise error [FWE]). Other statistical analyses were performed with Prism (GraphPad software, version 9.3.1.471). Two-way ANOVA followed with LSD Fisher’s post-hoc test was performed and p-value < 0.05 was retained. Correlation tests were made to determine Pearson’s correlation coefficient (r) and the p-value was obtained with Z test, and correlation matrix were done. The number of animals for each experiment is detailed in each figure legend.

## Results

### DBM reveals macrostructural changes and local tissue vulnerability to irradiation

To better understand radiation-induced cerebral atrophy previously described in this animal model, we analysed the brain macrostructure changes by DBM. As presented on Fig. 1A, the statistical differences (*p* < 0.01) were overlayed on rat brain template. The blue clusters indicate that Jacobian determinant (JD) values in irradiated group were significant inferior to those in control group, reflecting a shrinkage of brain tissue due to irradiation. Conversely, the red clusters denote that JD values in irradiated group were significant superior to those in control group, reflecting an expansion of brain tissue due to irradiation.

The results of voxel-wise comparison showed that WBI induced significant macrostructural alterations of the brain from 1 month up to 6 months after irradiation (Fig. 1A). At 1 month, mostly blue clusters were observed in the brain while at 3 and 6 months post-irradiation, red clusters dominated in the brain. Considering that the largest cluster could be located in different brain locations, we then focused with a zoom on each one (corresponding to the green box in Fig. 1A). Thus, we observed that the major macrostructural modifications highlighted by DBM were more pronounced at 1 month and 6 months following brain irradiation. Indeed, the proportion of significant voxels in the blue clusters was maximal 1 month after WBI since they represented 3.8% of the whole brain volume (Fig. 1B). On the other hand, the proportion of significant voxels in the red clusters increased in the late stages of WBI: they were equivalent to 5.5% and 7.6% of the whole-brain volume at 3 and 6 months post-WBI respectively (Fig. 1B).

Finally, the significant blue clusters (1 month after WBI) and red clusters (6 months after WBI) were registered on rat brain atlas to precisely identify the brain structures having major macrostructural modifications. As shown in Fig. 1C, the major blue clusters (DBM clusters-1 M) were localized in the corpus callosum which was locally contracted and the majority of red clusters (DBM clusters-6 M) were present in the cortex which showed local tissue expansion. These DBM-derived data indicate that the corpus callosum and the cortex are prone to radiation-induced tissue changes.

### WBI causes microstructural changes in brain structures identified as the most vulnerable by DBM

**Fig. 3:**
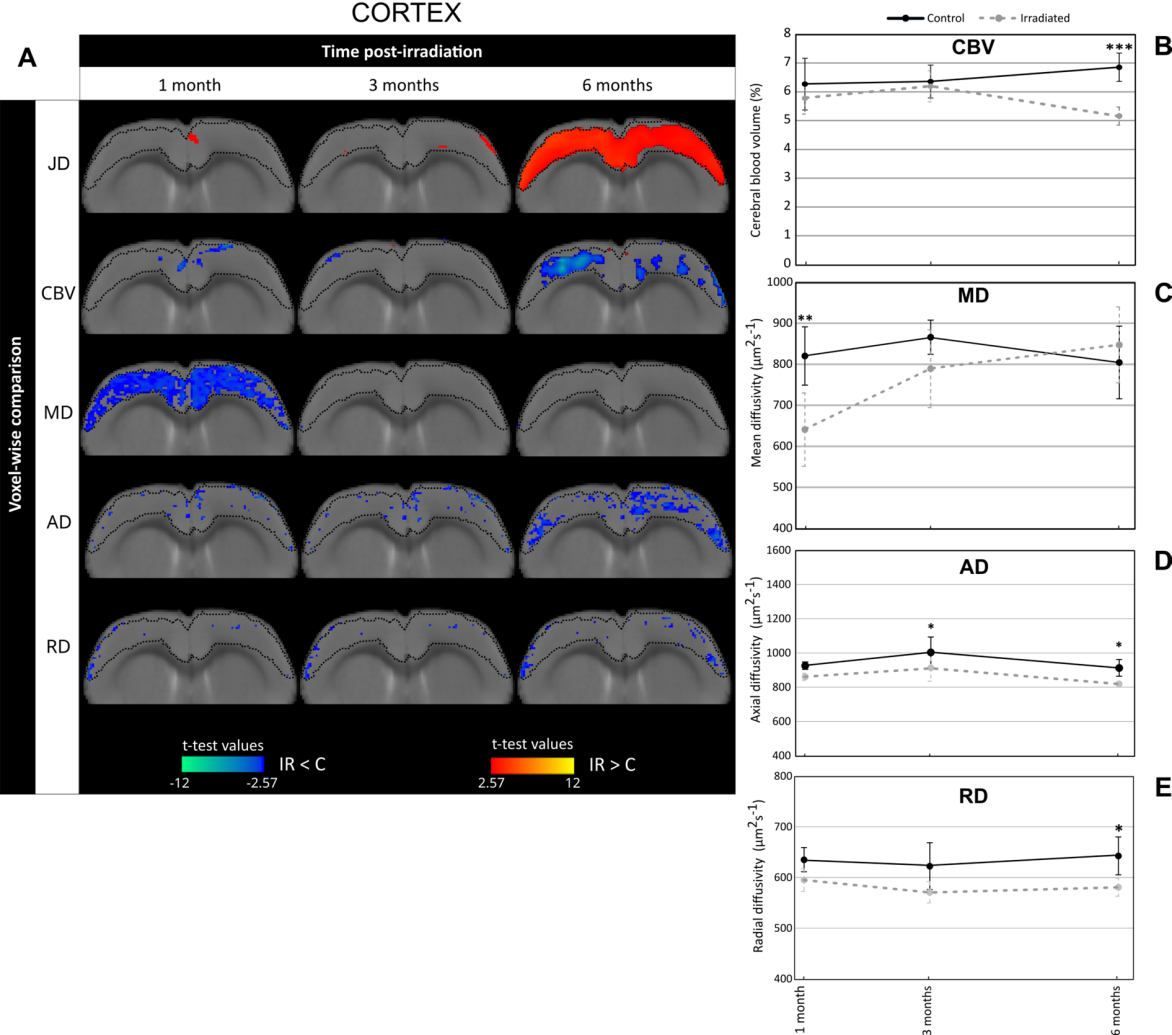
Irradiation induces changes in cerebral blood volume and diffusion MRI-derived parameters in the cortex. (**A**) Voxel-wise comparison of Jacobian determinant (JD), cerebral blood volume (CBV), mean diffusivity (MD), axial diffusivity (AD) and radial diffusivity (RD) in the cortex between control and irradiated groups (noted C and IR respectively) at different times after whole-brain irradiation (Student’s t-test with significant threshold set at *p* < 0.01). The macrostructural changes highlighted by DBM in the cortex at 6 months post-irradiation are demarcated on the T2w images by dotted lines. The statistically different voxels are located on the rat brain template (T2w image). The blue clusters correspond to significant t-test when MRI parameter values of irradiated group are lower than those of the control group. The red clusters correspond to significant t-test when MRI parameter values of irradiated group are higher than those of the control group. (**B-E**) Quantification of CBV (**B**), MD (**C**), AD (**D**) and RD (**E**) in the cortex at 1, 3 and 6 months after whole-brain irradiation. Black line corresponds to the control group and gray line to the irradiated group. The number of animals for the control group is *N* = 6 rats and for the irradiated group is *N* = 4–6 rats. Mean ± SD, two-way ANOVA (group and time effects) followed by Fisher’s LSD test: * *p* < 0.05, *** *p* < 0.001

To better understand the origin of DBM-revealed alterations in the brain of irradiated animals, we then assessed changes in tissue microstructure. For this, we studied cerebral blood volume (CBV) and MRI-derived diffusion parameters, namely mean diffusivity (MD), axial diffusivity (AD) and radial diffusivity (RD) in the brain areas including major significant clusters identified by DBM, i.e. in the corpus callosum (DBM clusters-1 M) (Fig. 2) and the cortex (DBM clusters-6 M) (Fig. 3).

In the corpus callosum region, the voxel-wise comparisons between irradiated and control groups showed that the blue clusters extracted from JD analysis (DBM clusters-1 M, indicated by dotted lines on all T2w images) were spatially associated to blue clusters for MD at 1 month after WBI. At 6 months post-irradiation, only a few blue clusters for RD were observed (Fig. 2A). These observations are sustained by quantifications of MRI parameters studied in corpus callosum (corresponding to DBM clusters-1 M) (Fig. 2B and E). Concerning CBV, irradiated rats had significant decreased values compared to control rats at only 6 months post-WBI (CBV_irradiated_ = 2.36 ± 1.2% and CBV_control_ = 3.20 ± 1.16%; *p* < 0.01) (Fig. 2B). In contrast MD values were significantly reduced in irradiated group relative to control one: MD_irradiated_ = 586.04 ± 47.95 μm².s^− 1^ and MD_control_ = 762.44 ± 41.40 μm².s^− 1^ (*p* < 0.01) (Fig. 2C). Whatever the time studied, the AD values were similar between the two animal groups (Fig. 2D) whereas WBI significantly diminished RD values in the corpus callosum at 3 months (*p* < 0.05) and 6 months post-irradiation (*p* < 0.01) (Fig. 2E).

In the cortex, the voxel-wise comparisons showed that the red clusters extracted from JD analysis (DBM clusters-6 M, indicated by dotted lines on all T2w images) were spatially associated to blue clusters for MD at 1 month after WBI and, to a lesser extent, to blue clusters for CBV and AD at 6 months after brain irradiation (Fig. 3A). The measurements of CBV values in the cortex confirm the voxel-wise comparison since CBV values significantly reduced in irradiated group compared to control group in the late phase (CBV_irradiated_ = 5.13 ± 2.15% and CBV_control_ = 6.83 ± 2.47%; *p* < 0.001) (Fig. 3B). Regarding MD, the values were significantly reduced in irradiated animals in comparison to control ones at 1 month after WBI: MD_irradiated_ = 629.55 ± 95.04 μm².s^− 1^ and MD_control_ = 820.70 ± 75.72 μm².s^− 1^ (*p* < 0.01) (Fig. 3C). For AD and RD parameters, irradiated animals displayed significant decreased values relative to control animals at 6 months post-WBI (*p* < 0.05) (Fig. 3D and E).

To confirm these data obtained by MRI, an immunohistology study was performed to visualize brain vessels, astrocytes and myelin by collagen-IV, anti-GFAP and anti-MBP immunostainings respectively. As presented in Additional Fig. 3A and B, the brain vessels were less numerous in irradiated animals compared to control ones at 6 months after WBI. Otherwise, to better understand the diffusion MRI data, we were interested to astrogliosis involved in neuroinflammation. The immunohistology images obtained with GFAP antibody showed that astrocytes in the irradiated cortex had a more stellate and branched shape compared to the control rats, which indicates that WBI induced astrogliosis (Additional Fig. 3C). Indeed, the immunostaining quantification evidenced that WBI led to a significant enhancement of the cortical surface occupied by astrocytes during the early phase (*p* < 0.001) (Additional Fig. 3D). We also observed both a reduction and a fragmentation of myelin around axons into the striatum (Additional Fig. 3E). The quantification of MBP positive surface from the immunostaining images confirmed that WBI significantly reduced myelin compared to non-irradiated animals at only 6 months post-irradiation (*p* < 0.001) (Additional Fig. 3F).

All of these results showed that the macrostructural changes evidenced by DBM were associated to tissue microstructural modifications as highlighted by MRI-derived diffusion and vascular parameters. Our data suggest that DBM changes could be linked to radiotoxicity phenomena such as neuroinflammation, vascular damage and demyelination.

### Relationships between the macrostructural alterations identified by DBM and the tissue microstructural changes induced by brain irradiation

**Fig. 4:**
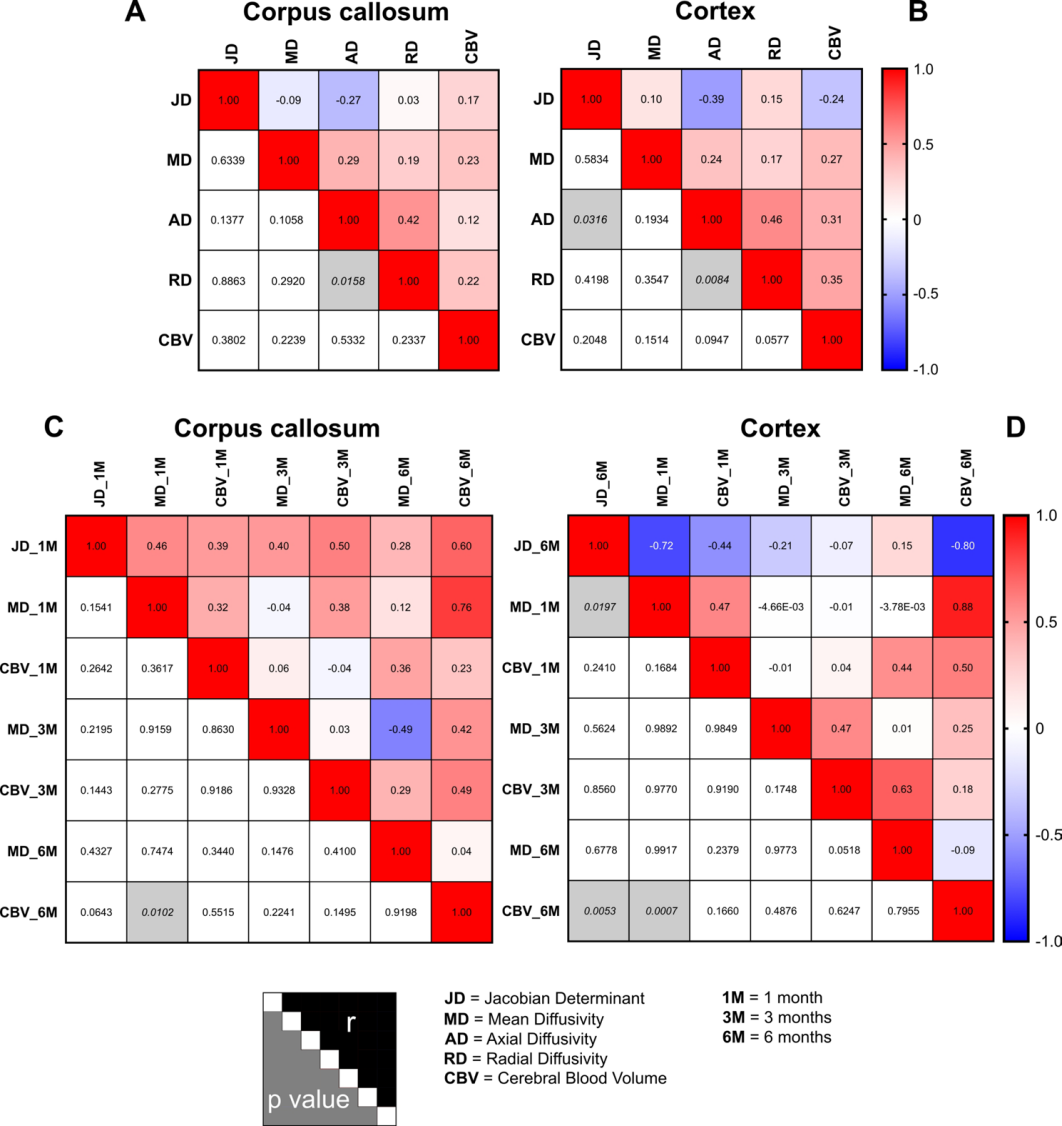
Correlations between MRI parameters in the brain structures identified by DBM: corpus callosum and cortex. (**A-B**) Correlation matrix by confounding both the time studied and the control and irradiated animals in the corpus callosum (**A**) and cortex (**B**). (**C**) Correlation matrix in the corpus callosum by focusing on Jacobian determinant measured at 1 month post-irradiation as a reference point. This specific time point was chosen for the correlation analyses due to the significant blue clusters identified in the DBM analysis that was found to be major in the corpus callosum. (**D**) Correlation matrix in the cortex by focusing on Jacobian determinant measured at 6 months post-brain irradiation as a reference point. This specific time point was chosen for the correlation analyses due to the significant red clusters identified in the DBM analysis that was found to be major in the cortex. For all correlation matrices, the coloured squares correspond to the Pearson’s correlation coefficient (r value) and the gray squares indicate a significant p-value. The correlations are performed between the MRI parameters studied: Jacobian determinant (JD), mean diffusivity (MD), axial diffusivity (AD), radial diffusivity (RD)

To go further on the interpretation of the results obtained with DBM, correlation analyses were performed to assess the potential relationships and temporal dependency between macrostructural changes (evaluated by JD) and microstructural changes (assessed by CBV, MD, AD, RD) induced by irradiation (Fig. 4).

The first analysis corresponds to the correlations between JD with CBV and diffusion metrics. In the corpus callosum, JD correlated neither with CBV nor diffusion metrics (Fig. 4A). Otherwise, the correlation matrix in the cortex showed only one significant correlation between JD and AD (*p* = 0.03) (Fig. 4B).

In a second correlation analysis, we elected to concentrate on the correlations between JD values obtained at 1 month (for corpus callosum) and 6 months (for cortex) with the two most clinically used MRI parameters, namely CBV and MD, at the different time studied. For correlation matrix obtained in the corpus callosum, even if most of Pearson’s coefficients were relatively close to 0.5, none p-values were significant. However, correlations were noted between JD and MD measured at 1 month (*r* = 0.46 and *p* = 0.15) as well as JD and CBV measured at 6 months (*r* = 0.60 and *p* = 0.06) (Fig. 4C). On the other hand, the matrix correlation performed in the cortex showed significant correlations between JD and MD measured at 1 month (*r* = -0.72 and *p* = 0.02) as well as JD and CBV measured at 6 months (*r* = − 0.80 and *p* = 0.005) (Fig. 4D).

Briefly, these results suggest DBM analysis evidenced local macrostructural alterations induced by brain irradiation could be associated to tissue microstructural changes, especially cell density or tissue composition in early phase (MD parameter) and vascular damage in late phase (CBV parameter).

## Discussion

Although RT is essential in the management of patients with brain tumors, it leads to cognitive declines that dramatically alter the quality of life of those treated patients [2, 3]. To improve the management of brain sequalae caused by RT, it is crucial to propose sensitive tools that detect irradiation-associated brain damage. In this context, MRI could be relevant [6]. Many clinical and preclinical studies clearly demonstrated that cranial RT induces brain atrophy, which appears to be dose dependent [28, 29]. Some clinical studies based on manual or semi-automatic delineations of brain structures evidenced the loss of cerebral tissue [30, 31]. However, to our knowledge, very rare studies have currently used computational and unsupervised morphometry based analyses to study radiation-induced brain damage [18, 19].

Here, we investigated the relevance of DBM approach to detect brain alterations and local vulnerability of cerebral tissue to irradiation. Based on the whole-brain irradiation in the rat, in which we previously showed a late radiation-induced global brain atrophy (about 6%) brain [20], we evidenced that DBM analysis enables to identify the local vulnerability of brain tissue to irradiation. Thus, we highlighted that the corpus callosum and the cortex are prone to radiation-induced tissue changes. We also demonstrated in this experimental model that macrostructural changes identified by DBM were associated to microstructural changes in brain cells and vessels, as evidenced by diffusion metrics and CBV changes. Finally, since the analysis of matrix correlations did not demonstrate a strong correlation between all MRI parameters studied, DBM has identified local macrostructural modifications that appear to be linked to early microstructural changes at the cellular level or later at the vascular level (with MD or CBV, respectively).

Based on the DBM approach, we observed local vulnerability of brain areas to irradiation. This vulnerability was first observed in the corpus callosum after 1 month post-WBI, and larger clusters were identified in the cortex at 3 and 6 months post-WBI. Interestingly, these cerebral structures have already been identified as sensitive to cranial RT in patients. Indeed, the cortex plays a significant role in cognitive processes (memory, attention, learning, executive functions) and many clinical studies demonstrated its vulnerability to RT [32]. Karunamuni and colleagues used high-resolution MRI to measure cortical volumetry and showed that patients with high grade glioma experienced dose-dependent cortical atrophy one year after RT [33]. One of the effects of RT is a dose-dependent thinning of the cerebral cortex, which may contribute to cognitive impairments after treatment [34]. The corpus callosum also plays a crucial role in cognition as it serves as the center of cerebral connectivity and the synchronization of the two cerebral hemispheres. It has been shown that cranial RT induces corpus callosum atrophy [35]. Furthermore, damage to this white matter structure has been linked to impaired cognitive control [36].

Indeed, the periventricular white matter is well known to be very sensitive to irradiation. White matter lesions usually develop around the periventricular white matter at the beginning and progress to diffuse white matter changes with varying degrees of cerebral atrophy over months or years [37, 38]. It could indeed be hypothesized that in our study that the region identified as “corpus callosum” also includes the periventricular white matter. However, the brain volume being relatively small in rats and given the spatial resolution of MRI, it is very difficult to identify the periventricular white matter in rodents. So, the animal model used in our study is not appropriate to this hypothesis, it would be appropriate to investigate by DBM approach the effects of radiation therapy in the healthy brain of the non-human primates in which the relative amount of white matter is greater than that of rodents [39, 40].

The JD analysis showed a shrinkage of the corpus callosum 1 month after radiation, which may underly cerebral atrophy. However, JD values in the cortex were higher in irradiated rats than in control rats, suggesting tissue expansion. This result could be better understood in line with a recent study showing that chemotherapy induced a volume expansion of white matter in breast cancer patients without any modification of the whole brain volume. The authors suggested that the extension of white matter may be due to a proinflammatory state in the brain. This process could be a volume response originating from a protective mechanism against the cerebral impact of chemotherapy [41].

To go further in the understanding of DBM analysis, we chose to investigate radiation-induced microstructure changes. Firstly, the analysis of brain vascularization by MRI showed that WBI led to a significant decrease in CBV values at 6 months post-irradiation. These MRI results were confirmed by immunohistology. Our data are in accordance with few preclinical studies which demonstrated the negative impact of cerebral irradiation on vascularisation [1, 5]. This is evidenced by a decrease in the number of endothelial cells, as well as reduction in the density and length of vessels. Clinical studies also demonstrated that cranial RT causes vascular damage, leading to aberrant morphology and dysfunction of the vessels. This could result in local cerebral ischemia, and in some cases, stroke has been observed as a side effect in patients [8]. Otherwise, diffusion MRI can be used to assess the cellular microstructure and the brain integrity. Cerebral irradiation resulted in MD decrease during the early phase (1 month post-irradiation) may be due to neuroinflammation linked to the activation of astrocytes and microglia. The hypothesis that radiation-induced neuroinflammation explains the decrease in MD values is partially supported by the presence of astrogliosis as demonstrated by the increase in GFAP immunostaining performed on the cortex. Similarly, WBI induced modifications in other diffusion parameters, particularly AD and RD, in our animal model. However, these observations were only noted during the late phase of brain irradiation and may be due to the presence of axonal degeneration/demyelination induced by brain irradiation as we evidenced by MBP immunostaining made into the striatum. The changes in MRI-derived diffusion parameters observed in gray or white matter are similar to those reported in the literature [42, 43]. A clinical study showed a decrease in MD, AD and RD values in the normal appearing white matter of glioma patients following RT. The authors attributed this to reactive astrogliosis, axonal degradation, axonal swelling, and resolving edema [44].

One main point of our study is the investigation of relationships between macrostructural and microstructural changes in the most vulnerable cerebral structures identified by DBM. We evidenced that local macrostructural alterations induced by brain irradiation could be associated to tissue microstructural changes, especially cell density or tissue composition in the early phase (JD/MD correlation) and vascular damage in the late phase (JB/CBV correlation). The causal relationships between macrostructural and microstructural modifications in the brain have been widely studied in recent years, particularly in aging and neurodegenerative diseases. However, it is difficult to conclude because the results are very controversial: some studies reported that the diffusion metrics and cerebral volume changes derived from JD values are congruent [45, 46] while others concluded that they are independent [47, 48]. Regarding a potential link between macrostructural changes and brain vascularization, there are few studies in the literature. Some reports suggest that cerebral atrophy measured using Jacobian determinants could be correlated with cerebral blood flow [49, 50]. Moreover, a recent study combined MRI analysis based to VBM and DBM mixed approach with two‑photon microscopy, performed in the mouse, brain volume changes are not strongly related to changes in physical volume, suggesting that the results of VBM/DBM cannot solely be interpreted as mere changes in tissue volume. These authors purpose that many possible mechanisms within the tissue may influence the cerebral volume such as local cell density, spatial arrangement of cells as well as cell type composition [51].

Few clinical studies applied to brain tumor patients using DBM approach from MRI images indicate that quantitative morphometric monitoring associated to radiation-induced brain injury is feasible [18, 31, 49]. In these studies, tumor beds and the surrounding tissues were masked and censored during the analysis to take into account any tumor-associated morphological changes between MRI scans. This step prevents errors due to tissue misclassification at the tumor bed and in the surrounding tissues, and makes sure that the observed morphological changes are related to the applied radiation. Moreover, it has been also proposed that features extracted from the Jacobian map is able to quantify local tumor morphological changes using only baseline tumor contour without post-treatment tumor segmentation. Jacobian maps derived from DBM approach showed great potential for longitudinal evaluation of tumor response [52]. On the other hand, VBM approach has also recently been used to evaluate the radiotherapy in patients with gliomas [30, 53]. In these studies, the tumor-containing hemisphere was masked during all VBM analyses performed with SPM because this software is unable to correctly identify and segment gray matter and white matter in the proximity of tumors. Together with the selective flipping, this process ensures that the “tumor-free” hemisphere can be reliably analyzed. Finally, all of these studies conducted on patients with brain tumors demonstrate the feasibility to use DBM approach to evaluate radiation therapy toxicities on brain tissue but also to determine the tumor response to anticancer treatments. Nevertheless, the choice of software and the different associated metrics should be validated before a routine application. To go further, preclinical studies on animals bearing brain tumors will be considered in order to challenge the DBM approach for evaluation of radiation effects on the normal brain tissue over time.

VBM and DBM used for morphometry analysis rely on a fully automated method, but they differ in many aspects. The DBM do not use a segmented image contrary to VBM, so the whole information in grayscale is considered, and not only gray matter. Then, the DBM is a registration in 3 spatial dimensions unlike VBM and only DBM permit to do longitudinal comparisons for a same animal or human. The anatomic differences are also kept in deformations fields and it is possible to detect more subtle structural differences with DBM than VBM. In contrast, VBM is freely available for most major neuroimaging software (SPM and FSL software), unlike DBM for which automated software is few. The ANTs software that is most used to performed DBM, is developed under significant open-source infrastructure to facilitate reproducible research and includes significant documentation, working examples, and scripts used in previous successful studies [54]. One of the main advantages of DMB is its possibility of being performed on T1 or T2 images from different research laboratories or medical centers. Thus DBM can be successfully applied to a number of multi-site projects [12]. Moreover, the mathematical model of DBM using JD is already used by radiation oncologists for the dosimetry performed on CT-scan as part of the radiotherapy protocol, an approach known under the term “Deformable image registration”. This approach has also been suggested to evaluate cerebral radiotoxicity [55].

One of the limitations of our study is the lack of behavioral data and its correlation with DBM results. In contexts other than radiation-induced neurotoxicity, correlations between JD and cognition have been highlighted [56, 57] and it has even been proposed that DBM could be relevant to predict the cognitive deficits in Alzheimer’s disease [58]. Unfortunately, we were unable to conduct an MRI study and behavior analyses on the same animals due to the complexity of the experimental protocol. Future studies are necessary to determine whether the Jacobian determinants quantified with DBM are correlated with cognitive deficits as well as their predictive values.

## Conclusions

Taken together, all the results evidence that brain irradiation induced local macrostructural changes detected by DBM, which are associated to tissue microstructural modifications as highlighted by MRI-derived diffusion and vascular parameters. Our data suggest that DBM changes could be linked to radiotoxicity phenomena such as neuroinflammation and vascularization alterations. Moreover, this computational morphometry approach appears to be relevant to investigate brain vulnerability to irradiation. Although further preclinical investigations are necessary to determine if there is a correlation between DBM and cognitive deficits, the transposition of these findings to patients may provide added value in imaging studies on cerebral radiotoxicity.

### Electronic supplementary material

Below is the link to the electronic supplementary material.

Supplementary Material 1

Supplementary Material 2

Supplementary Material 3

## Data Availability

Research data are stored in an institutional repository and will be shared upon reasonable request to the corresponding author.

## Abbreviations

AD: Axial diffusivity
ANTs: Advanced Normalization Tools
BSA: Bovine serum albumin
CBV: Cerebral blood volume
CSF: Cerebrospinal fluid
DBM: Deformation-based morphometry
DTI: Diffusion tensor imaging
FEW: Family-wise error
GE-EPI: Gradient echo-echo planar imaging
GM: Gray matter
JD: Jacobian determinant
MD: Mean diffusivity
MRI: Magnetic resonance imaging
PBS: Phosphate buffered saline
RD: Radial diffusivity
RT: Radiotherapy
SE-EPI: Single shot-echo planar imaging
VBM: Voxel-based morphometry
WBI: Whole-brain irradiation
WM: White matter
