# Supplementary material for: Deformation-based morphometry: a sensitive imaging approach to detect radiation-induced brain injury?
Source: Cancer Imaging. 2024 Jul 18;24:95. doi: 10.1186/s40644-024-00736-1 (PMC11256482; doi:10.1186/s40644-024-00736-1)
Supplement: Supplementary file 1 — Supplementary Material 1 [file 40644_2024_736_MOESM1_ESM.docx]

**
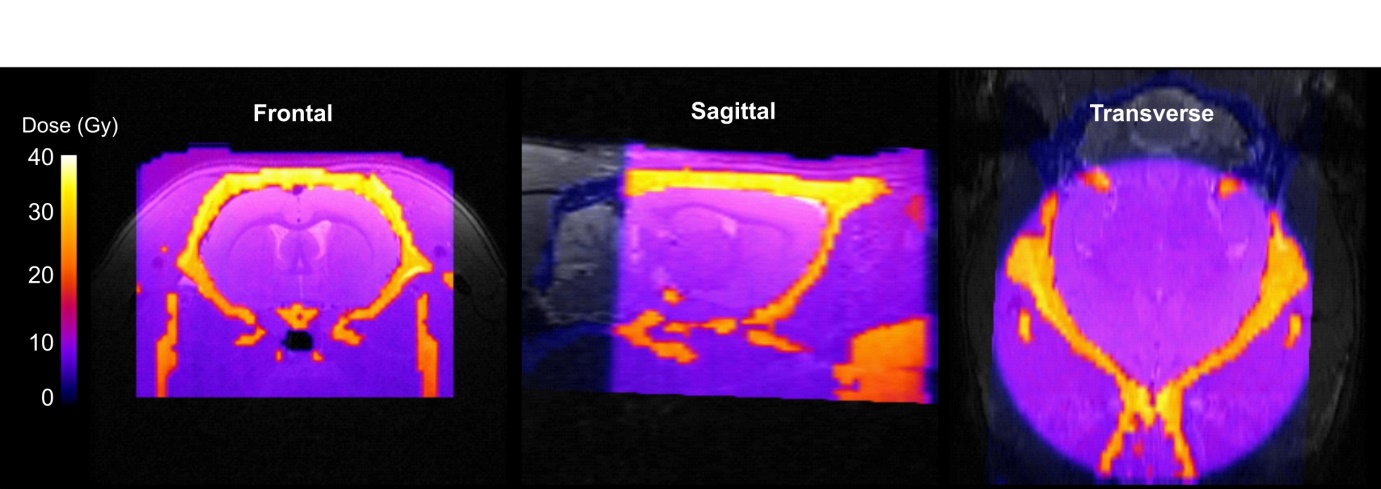
**

**Additional Fig. 1: X-ray dose deposition map in the whole brain**

The dose deposition map, generated by Monte Carlo simulations from the treatment planning system (TPS), for a single fraction of irradiation (10 Gy) was registered on anatomical MRI images (T2-weighted images). The whole-brain irradiation procedure used showed a spatial homogeneity of irradiation in all the brain structures
