# Supplementary material for: Deformation-based morphometry: a sensitive imaging approach to detect radiation-induced brain injury?
Source: Cancer Imaging. 2024 Jul 18;24:95. doi: 10.1186/s40644-024-00736-1 (PMC11256482; doi:10.1186/s40644-024-00736-1)
Supplement: Supplementary file 2 — Supplementary Material 2 [file 40644_2024_736_MOESM2_ESM.docx]

**
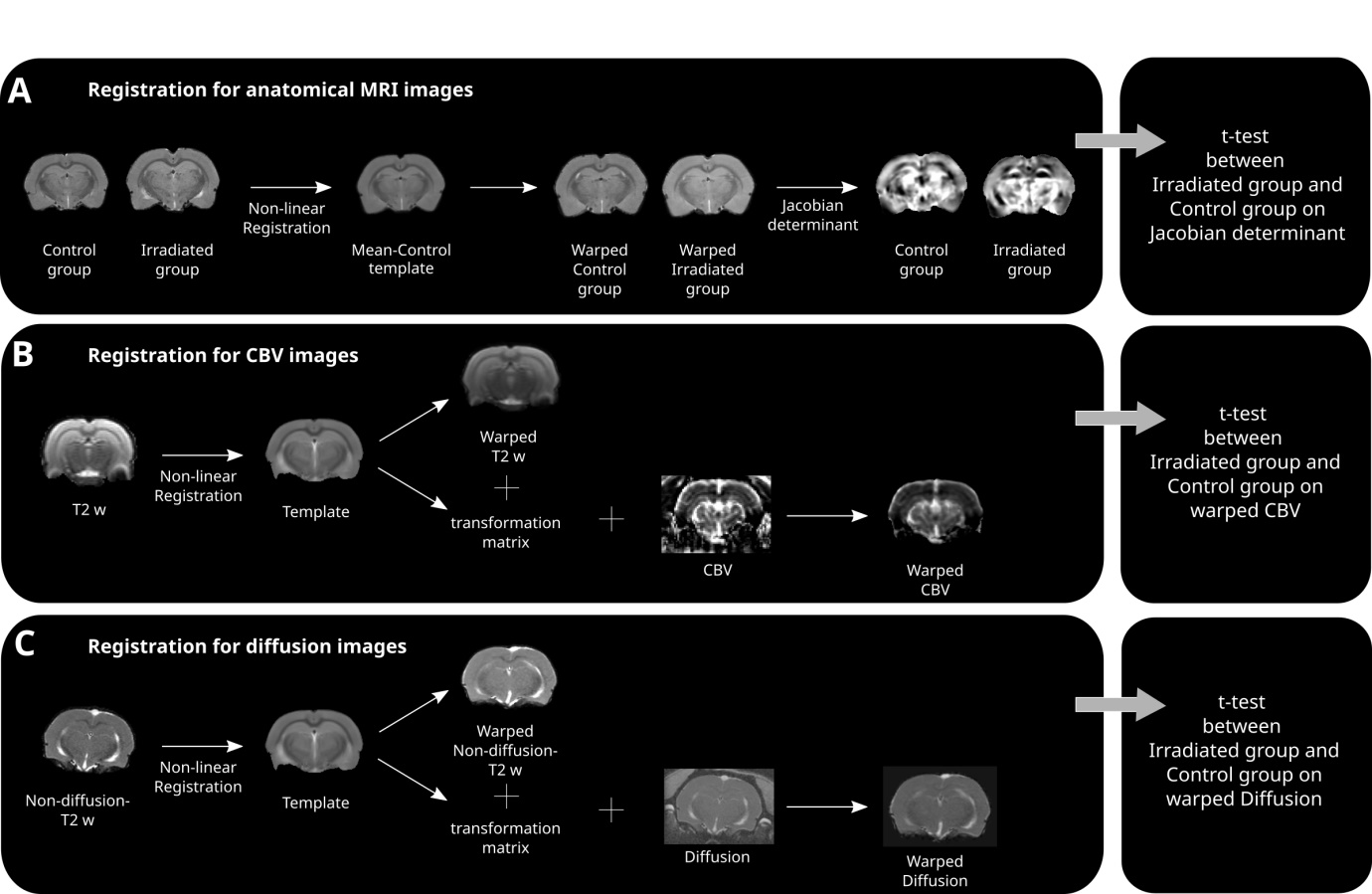
**

**Additional Fig. 2: Registration procedures for MRI images**

**(A)** Registration of anatomical MRI images on a Mean-Control template defined for each time point after brain irradiation. Construction of Jacobian determinant maps reflecting the local deformations in the brain necessary during registration of T2w images on Mean-Control template

**(B)** Registration of CBV images on the rat brain template (the same reference template was used for all the time studied). The first step involves registering T2w images onto the template (at the same resolution as the T2* images used to generate cerebral blood volume (CBV) maps). The second step involves applying the transformation matrix to the CBV maps

**(C)** Registration of diffusion images on the rat brain template (the same reference template was used for all the time studied). The first step involves registering non-diffusion T2w images, also known as A0 images, with a b value of 0 s.mm^− 2^, onto the template. The second step involves applying the transformation matrix to the diffusion metrics maps: mean diffusivity (MD), axial diffusivity (AD) and radial diffusivity (RD)
