# Supplementary material for: Deformation-based morphometry: a sensitive imaging approach to detect radiation-induced brain injury?
Source: Cancer Imaging. 2024 Jul 18;24:95. doi: 10.1186/s40644-024-00736-1 (PMC11256482; doi:10.1186/s40644-024-00736-1)
Supplement: Supplementary file 3 — Supplementary Material 3 [file 40644_2024_736_MOESM3_ESM.docx]

**
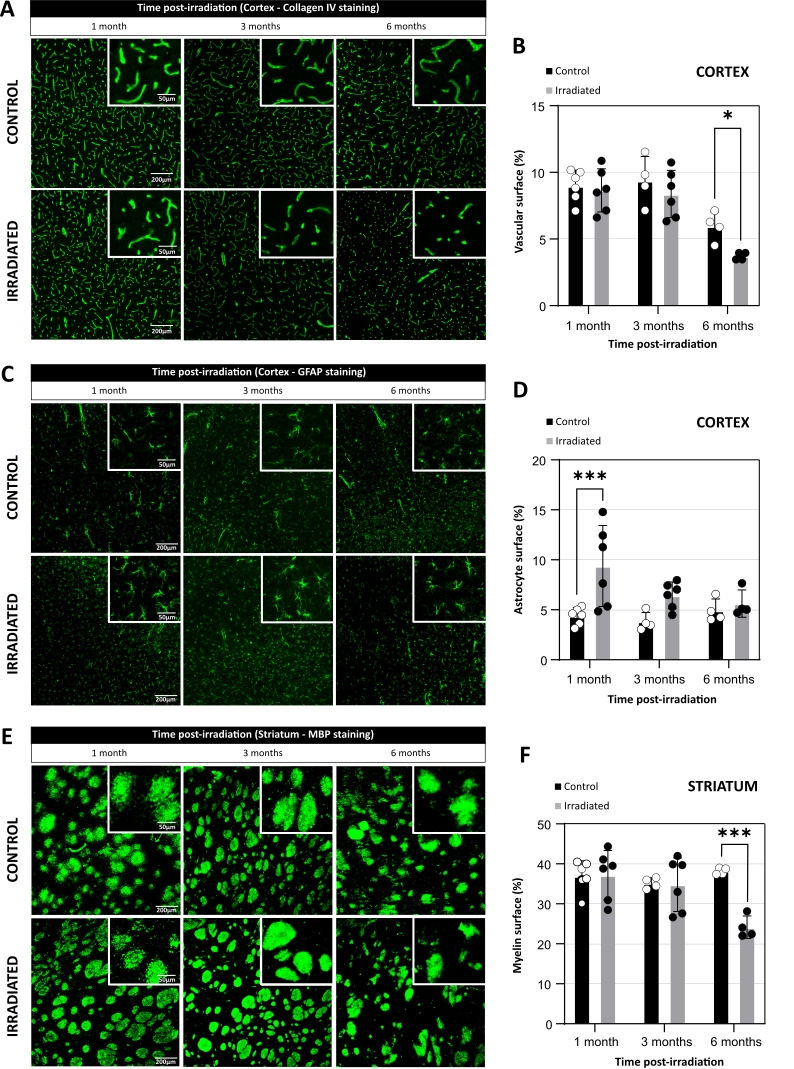
**

**Additional Fig. 3: Immunohistology analysis of blood vessels and astrocytes in the cortex at different times after irradiation**

**(A)** Representative images of immunostainings performed with anti-collagen IV antibody (in green) revealing blood vessels in the cortex of control and irradiated rats at 1, 3 and 6 months post-irradiation. Acquisition by fluorescence microscopy with a 10X objective; scale bar = 200 μm (full image) and 50 μm (enlarged image)

**(B)** Quantification of surface stained by vessels (%). Mean ± SD; N = 4–6 rats/group. Two-way ANOVA (group and time effects) followed by Fisher’s LSD test: * p < 0.05

**(C)** Representative images of immunostainings performed with anti-glial fibrillary acidic protein antibody (GFAP, in green) revealing reactive astrocytes in the cortex of control and irradiated rats at 1, 3 and 6 months post-irradiation. Acquisition by fluorescence microscopy with a 10X objective; scale bar = 200 μm (full image) and 50 μm (enlarged image)

**(D)** Quantification of surface stained by astrocytes (%). Mean ± SD; N = 4–6 rats/group. Two-way ANOVA (group and time effects) followed by Fisher’s LSD test: *** p < 0.001

**(E)** Representative images of immunostainings performed with anti-myelin basic protein antibody (MBP, in green) revealing myelin in the striatum of control and irradiated rats at 1, 3 and 6 months post-irradiation. Acquisition by fluorescence microscopy with a 10X objective; scale bar = 200 μm (full image) and 50 μm (enlarged image)

**(F)** Quantification of surface stained by myelin (%). Mean ± SD; N = 4–6 rats/group. Two-way ANOVA (group and time effects) followed by Fisher’s LSD test: *** p < 0.001
